# Development of Novel Bioreactor Control Systems Based on Smart Sensors and Actuators

**DOI:** 10.3389/fbioe.2020.00007

**Published:** 2020-02-04

**Authors:** Baowei Wang, Zhiwen Wang, Tao Chen, Xueming Zhao

**Affiliations:** ^1^Department of Biochemical Engineering, School of Chemical Engineering and Technology, Tianjin University, Tianjin, China; ^2^SynBio Research Platform, Collaborative Innovation Center of Chemical Science and Engineering (Tianjin), Tianjin, China; ^3^Frontier Science Center for Synthetic Biology and Key Laboratory of Systems Bioengineering (MOE), School of Chemical Engineering and Technology, Tianjin University, Tianjin, China

**Keywords:** flat organizational control systems, knowledge-based control systems, bioreactors, smart sensors, actuators

## Abstract

Bioreactors of various forms have been widely used in environmental protection, healthcare, industrial biotechnology, and space exploration. Robust demand in the field stimulated the development of novel designs of bioreactor geometries and process control strategies and the evolution of the physical structure of the control system. After the introduction of digital computers to bioreactor process control, a hierarchical structure control system (HSCS) for bioreactors has become the dominant physical structure, having high efficiency and robustness. However, inherent drawbacks of the HSCS for bioreactors have produced a need for a more consolidated solution of the control system. With the fast progress in sensors, machinery, and information technology, the development of a flat organizational control system (FOCS) for bioreactors based on parallel distributed smart sensors and actuators may provide a more concise solution for process control in bioreactors. Here, we review the evolution of the physical structure of bioreactor control systems and discuss the properties of the novel FOCS for bioreactors and related smart sensors and actuators and their application circumstances, with the hope of further improving the efficiency, robustness, and economics of bioprocess control.

## Introduction

The history of bioreactor development is as old as the use of microorganisms by ancient cultures to ferment and improve foods and beverages. Since the invention of submerged fermentation, bioreactors have found wide applications in diverse fields including wastewater treatment in the environmental protection sector, cell culture and tissue engineering in the healthcare sector, the production of high-value pharmaceuticals and bulk chemicals in industrial biotechnology, and even the cultivation of algae for oxygen generation in space exploration (Li X. et al., 2016; Pirasaci et al., 2017; Zhuo et al., 2018; Christoffersson and Mandenius, 2019). Strong demand for various applications stimulated progress in bioreactor structure design to fulfill specific purposes, such as solid-state fermentation bioreactors used in the Chinese alcoholic beverage (Baijiu) brewing industry, anaerobic membrane bioreactors for wastewater treatment, classic tank bioreactors used in the fermentation industry, and the recently developed inexpensive single-use bioreactors for the small-scale production of high-value biological pharmaceuticals (Zhuo et al., 2018; Fang et al., 2019; Metze et al., 2019).

**FIGURE 1:**
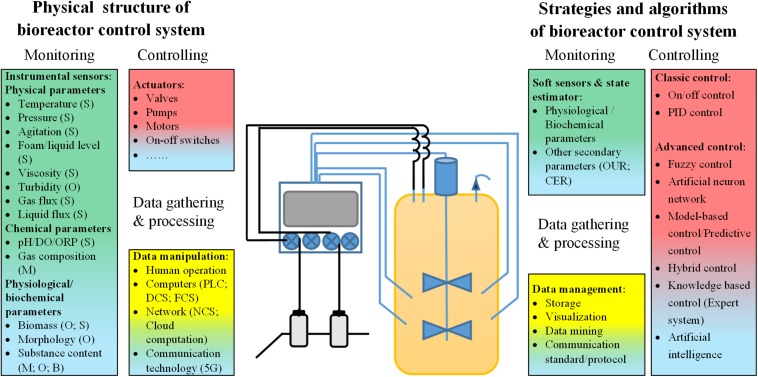
Summary of research on bioreactor control systems. S, specific functional sensors; O, optical sensors; M, MS-based online detectors; B, biosensors; DO, dissolved oxygen; ORP, oxidation-reduction potential; PLC, programmable logic controller; DCS, distributed control system; FCS, fieldbus control system; NCS, network control system; OUR: oxygen utilization rate; CER, CO_2_ emission rate; PID control, proportion-integral-differential control.

In addition to the development of new bioreactor geometry designs, the corresponding control systems have also played an important role in the development of bioreactors. In general, the main functions of a bioreactor control system include process control, monitoring, data gathering, and processing. The current research on bioreactor control systems can be grouped into two aspects, with some studies focusing on control strategies and algorithms adapted to bioreactor control systems, while others mainly investigate the physical structure of the instrumental organization of bioreactor control systems (Figure 1). Research on the control strategies and algorithms adopted in bioreactor control systems is mainly focused on problems related to monitoring parameters that cannot be directly measured, methods of effective and stable control based on the monitoring of process data, and the successful application of process data to improve future processes (such as dynamic bioprocess modeling). Significant progress was also made in the development of control strategies, novel optimization algorithms, and software frameworks for control systems. These topics have been the subject of other excellent reviews (Simutis and Lubbert, 2015; Narayanan et al., 2019; Steinwandter et al., 2019; Steinwandter and Herwig, 2019) and will not be discussed in depth here. Research on the physical structure of bioreactor control systems mainly deals with issues related to the development and application of novel sensors for measuring physical, chemical and physiological process parameters, actuators used in process control, and facilities for data gathering and processing. Along with the application of digital computers in bioreactor process control and the emergence of cutting-edge control strategies and algorithms, several important physical structures of bioreactor control systems have been developed. The first is the classic HSCS, which has become the dominant physical structure of bioreactor control systems since its invention (Rolf et al., 1982). Subsequently, novel fieldbus control and NCSs were developed, which can both be described as FOCSs, followed by the recently developed KBCS (Kohout et al., 2015; Van Loon, 2015; Ibrahim et al., 2018). Together with the application of smart sensors and actuators, these novel bioreactor control systems could have a profound impact on bioprocess control in the near future.

Here, we focus our discussion on the development of novel bioreactor control systems based on parallel distributed smart sensors and actuators that have a lower order of hierarchy and higher control efficiency. Functions of the industrial field computers in classic HSCS have been redistributed to smart sensors, actuators, and mobile terminals of upper-layer computers in these novel control systems. Fast progress in the development of novel smart sensors, mechanical engineering, and information and communication technology has the potential to make these novel bioreactor control systems more efficient, robust, and economical.

## Evolution of the Physical Structure of Bioreactor Control Systems

### Classic Hierarchical Structure Control Systems

Early bioreactor control was dependent on human operation based on workers’ knowledge and long-term practical experience. With the development of sensor technology, which enables on-line monitoring of various process parameters and the application of digital computers and actuators, process monitoring and control of bioreactors has stepped into the age of automation (Stanke and Hitzmann, 2013; Lemoine et al., 2017; Bockisch et al., 2019). Simultaneously, the HSCS for bioreactors emerged and evolved after decades of practical application (Rolf et al., 1982). From a technical perspective, the HSCS for bioreactors was developed in two stages, the PLC stage and the DCS stage.

**FIGURE 2:**
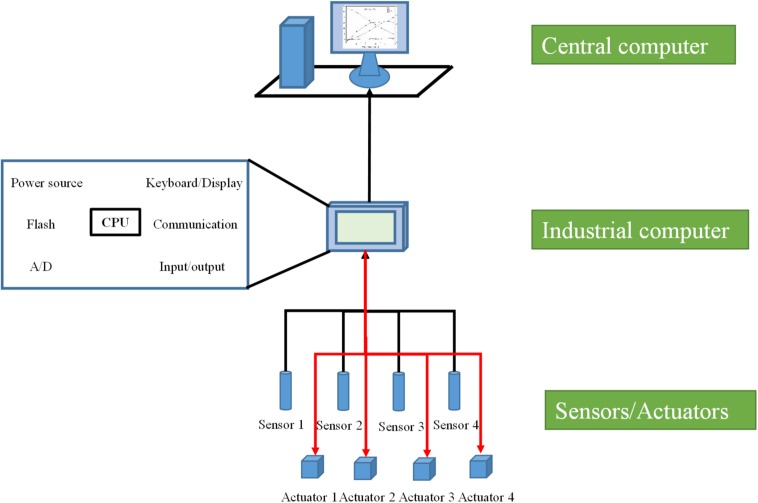
Classic hierarchical structure control system for bioreactors.

A classic bioreactor HSCS usually includes three layers (Figure 2). The bottom layer consists of process parameter monitoring and control devices (Rolf et al., 1982; Stanke and Hitzmann, 2013). In fermentation bioreactors, process parameters usually include physical, chemical, physiological, and biochemical parameters. For some physical and chemical parameters like temperature, pH, rotation speed, DO, pressure, liquid level, OD, and viscosity, specific functional sensors have been developed, and control of these process parameters can be carried out via classic control strategies based on specific actuators (Rodríguez-Duran et al., 2001). For physiological and biochemical parameters like biomass, key nutrient and metabolite concentrations, and gas composition, more complex monitoring facilities, as well as advanced control strategies and algorithms, are usually adopted (Lemoine et al., 2017; Bockisch et al., 2019). The middle layer of a bioreactor HSCS consists of industrial field computers, such as the early PLC systems with a microprocessor module. The functions of the middle layer comprise acquisition of the analogous signals from sensors and transforming them into digital signals (in some applications), recording process data, setting process parameters, running the optimizing algorithms for parameter control, generating actuation orders for actuators, presenting process data, and communicating with the upper-layer computers or other PLC systems. The middle-layer industrial field computer has served as a bridge for bidirectional data communication in HSCSs for bioreactors. The upper layer of a bioreactor HSCS consists of central computers such as a desktop computer. The main functions of the upper-layer central computers comprise acquiring process data from the industrial field computers and data management (e.g., visualization of process parameter data for real-time administration and comparing and analyzing historical data of recorded experiments for process evaluation and development). The upper-layer central computer is the highest terminal of a single operational unit of a bioreactor control system, but many upper-layer central computers can be connected via a local area network to form a larger control system like that of DCS for plants with large numbers of bioreactor units.

The classic HSCS for bioreactors represents tremendous progress compared to former distributed control and distributed administration and has been applied in commercialized bioreactors of different scales and complexities. The basic level usually incorporates personal computers equipped with a user-friendly OS and commercially available (e.g., LabView) or bioreactor supplier-provided software packages for controlling bench-top bioreactors. This arrangement provides a highly flexible platform for DIY systems and the exploration of new analytical and control devices. The middle level is a more highly integrated system that usually incorporates PLCs, a Human Machine Interface (e.g., a touchscreen) and a data recording module. In principle, fermentor systems provided by the major current suppliers (e.g., Biostat, BioFlo, Minifors, and Biobundle) can all be grouped into this category. The advanced level has been mainly used in pilot plants and production facilities (e.g., Eli Lilly and Company), which usually adopt DCS and may also be integrated with a PAT system, high-end data management, and MES for process monitoring and control (Alford, 2006).

However, there are also inherent drawbacks in the classic HSCS for bioreactors as compared with the newly developed FOCS (Table 1). Firstly, parameter signals from various sensors were separately sent to the middle layer industrial field computers and actuation orders from middle-layer computers to actuators point-to-point for process parameter control, so that extensive wiring and communication networks were required. Secondly, devices from different manufacturers could not be exchanged conveniently. Thirdly, from a functional perspective, the data acquisition, presentation, and analysis functions of the upper-layer central computers largely overlap with those of the middle-layer industrial field computers, especially with the fast progress in the computation capacity of microprocessors and storage capacity of flash chips. Finally, the rapid development of smart devices enabled the integration of signal processing and computation by smart sensors (Mora-Mora et al., 2015; Shabha and Conway, 2016; Wang et al., 2017; Lewis et al., 2018). Novel control systems of bioreactors that may avoid these drawbacks are very promising.

**TABLE 1:** Summary of the main characteristics of different types of control systems for bioreactors.

**Type**	**Controller**	**Monitor**	**Main characteristic**	**Drawback**	**Strength**
Pre-digital	– Human operation	– Human observation	– Distributed control and distributed administration	– Human error	– Low capital investment
				– High variability	– Lower maintenance cost
				– Less automation	
				– Low efficiency	
HSCS^a^	– Microcomputers	– Analog/digital detectors	– Distributed control and central administration	– High capital investment and maintenance cost	– High automation
			– Point-to-point communication of signals	– Complex wiring	– High efficiency
				– Function redundancy	– Complex control strategy
				– Low interoperability	
FOCS^b^	– Computers	– Digital detectors	– Distributed control and central administration	–Communication constraint of fieldbus	– Simplicity, accuracy, low maintenance cost
FCS^c^	– Smart devices	– Smart sensors	– Signals communication via fieldbus	– Signal delay and packet loss	– High interoperability, stability
					– Complex control strategy
NCS^d^	– Networked computers	– Digital detectors	– Distributed control and central administration	– Communication constraint of network	– High efficiency, low maintenance cost
	– Smart devices	– Smart sensors	– Signals communication via network	– Signal delay and packet loss	– High interoperability, stability
				– Risk of cyber-attack	– Advanced management

*^*a*^Hierarchical structure control system, including PLC and DCS systems; ^*b*^Flat organizational control system; ^*c*^Fieldbus control system; ^*d*^Network control system.*

### Fieldbus Control System

To overcome the drawbacks of the classic HSCS for bioreactors, researchers developed new strategies for building efficient and robust control systems. The FCS, which has been widely applied in many manufacturing processes, is the first step toward a novel FOCS for bioreactors (Leite et al., 2010; Van Loon, 2015). The FCS is discussed here as an alternative to the classical HSCS for bioreactors.

**FIGURE 3:**
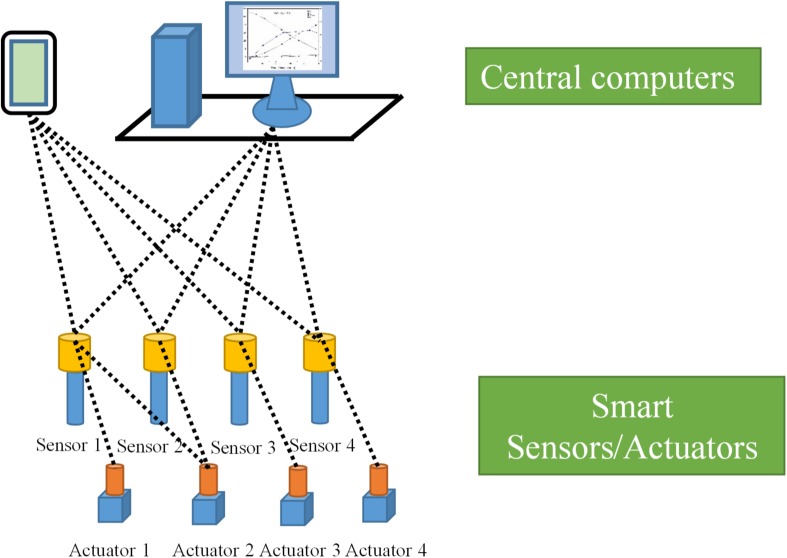
Representative structure of a novel flat organizational control system for bioreactors.

The most important feature of FCS design is the integration of separately distributed point-to-point signal transfer channels between field devices and controllers into one common communication channel, and, most of the time, digital signals instead of analog signals are transferred in the FCS according to specific communication standards. All field devices have been connected to the central computer via a fieldbus in the FCS (Verhappen, 2009). The FCS has taken the first step toward the development of a novel FOCS for bioreactors. Through the said design, the functions of process parameter monitoring and actuation, signal processing, data acquisition, and presentation belonging to different layers of the classic HSCS have been redistributed between the sensors/actuators, signal processing units and computation units of the novel FOCS for bioreactors. A scheme of the novel FOCS for bioreactors based on smart sensors/actuators connected to central computers via wireless communication is shown in Figure 3.

The physical architecture of the FCS can be divided into two layers, the first consisting of field devices such as sensors, actuators, and the communication channel (fieldbus) and the second comprising the central computer and its extended connections (Verhappen, 2009). Layer 1 of an advanced FCS consists of interoperable smart sensors and actuators. A smart sensor is an advanced sensing system that combines a sensing element with sensor signal processing and data computation capabilities provided by a microprocessor and related components (Gary et al., 2010). Accordingly, a smart actuator is referred to as an advanced actuator device, which combines the actuator element with a microprocessor and related components providing the actuation signal communication, computation, status diagnosing, and logging functions. These smart devices have *in situ* computation, self-diagnosing, rectifying, and operation history logging functions that may increase the integrity of the control systems, may reduce the time taken to diagnose process failure, and can be widely used in bioprocess regulation and control. The smart sensors and actuators form a closed-loop control of parameters according to the configurations of FCS through the sensor-actuator pairs. For more complicated parameter control, smart sensors may communicate with more than one actuator. In addition to the closed-loop control of specific process parameters, the smart sensors and actuators can also receive parameter configurations from the central computers and report process data to them. The common communication channel in each segment of an FCS has the function of bidirectional transfer of digital signals between the field devices and the central computers. During the past decades of FCS development, more than eight communication standards have been developed by different commercial organizations for effective communication between devices from different suppliers [Foundation Fieldbus (H-1); Controlnet; Profibus - DP, PA, and FMS; P-Net; HSE (High-Speed Ethernet) H-2; Swiftnet; WorldFIP; Interbus-S] (Verhappen, 2009). With the fast development of mobile communication technology, wireless communication has also been adopted for FCS applications (Zhu et al., 2012). Layer 2 of an FCS consists of central computers, and it is similar to the upper-layer computers of the classic HSCS for bioreactors. Instead of acquiring process data from middle-layer industrial field computers, the central computers of an FCS communicate process data with smart sensors directly via the fieldbus communication channel. In other words, the middle-layer field computer has been removed in the FCS. Data from layer 1 smart sensors and actuators acquired by central computers (through the host H1 card in case of the Foundation Fieldbus control system) can be presented in various forms and processed to display higher-order parameters for process evaluation. Mobile terminals of the central computer are also available for convenient process parameter configuration and status monitoring of bioreactors in production plants.

Because the point-to-point signal transfer in the classic HSCS for bioreactors has been replaced by fieldbus communication channels in FCS, the safety and reliability of the fieldbus communication channels have become vital topics in FCS development. Many strategies have been developed to meet the demands resulting from specific implementations (Zhong and Chen, 2015; Julsereewong et al., 2018). A paper by Zhong and Chen (2015) reported an effective method of integrating different fieldbus devices into one control system, which solved the problem of the stability of large FCSs. In more recent work, Julsereewong et al. (2018) conducted a comparative analysis of the safety problem of “control in the host” and “control in the field” in a Foundation Fieldbus (FF) control system, which could be used as an evaluation tool for the design phase of FF-based control loops. These related studies greatly improved the safety and stability of FCSs.

FCSs have already been successfully applied in bioreactor control in several studies. Wu and colleagues successfully adopted an FCS for the process control of L-asparaginase II fermentation that increased the output by 100% compared to conventional control methods (Xiaopeng and Baoguo, 2006). In addition to fermentation processes, an FCS has also been successfully implemented in process control of enzyme recovery plants for the evaluation of different intelligent controllers, and the experimental results confirmed the greater effectiveness of fuzzy controllers in comparison to neural predictive control. Moreover, the fuzzy PI controller exhibited reduced error, lower power consumption, and better recovery of enzyme activity (Leite et al., 2010). Van Loon (2015) reported that the application of intelligent FCS instead of compressed air lines in the control cabinets of the beverage manufacturer Teisseire increased the reliability and efficiency of its production line.

### Networked Control System

A NCS is another form of FOCS that can be used for bioreactor control. Just after the emergence of FCSs in the 1990s, the concept of connecting all field devices into a network and using the widely accessible internet for data transmission instead of special fieldbus systems restricted to specific communication standards had already been brought up. Investigation of NCSs has been a very hot topic since their appearance, and many detailed reviews have covered this field (Gupta and Chow, 2010; Mahmoud and IEEE, 2014; Marie et al., 2016). Here, we only added a brief discussion of this topic and its suggested future applications in bioreactor control.

While generally similar, the physical architecture of an NCS has one radical difference from that of an FCS: the hierarchical structure has been replaced by a fully distributed structure, with every device in the field having become a functional node in the network (Marie et al., 2016). Theoretically, all devices in the NCS network can communicate with each other under specific schedules. In a classical NCS, process parameter signals are converted to digital signals by sensors and sent to controllers, after which action instructions are sent from controllers to actuators, and the feedback control loop has been implemented through a network. This NCS architecture has made field control system data easily accessible from remote places and removed the information island of specific field devices and even specific control networks. Using the internet for data communication in the field control system has the advantages of high speed, convenient accessibility, and interoperability of devices while also enabling the implementation of more complicated control strategies (Marie et al., 2016).

NCS has been introduced in diverse fields, including precision agriculture, advanced manufacturing, energy, and transportation. In precision agriculture, the NCS has been used for artificial environment stabilization of greenhouses and the control of irrigation canal systems (Lai et al., 2015; Ibrahim et al., 2018). The NCS has also been widely applied in advanced manufacturing, including the control of mechanical factories (Cuenca et al., 2018). The application of NCS-based power system stabilizers in power grids has also been reported by Zhang et al. (2014). In the field of transportation, the NCS has been used for vehicle control and the management of highway traffic safety (Bao et al., 2017; Latrech et al., 2018). However, the use of an NCS for bioreactor control is still on the horizon, and few papers have dealt with this topic. As fermentations are always complicated and sensitive multi-parameter processes, precise and automatic control of process parameters is very important, indicating that the application of NCS for bioreactor control would be very promising.

In the NCS, the incorporation of a network in the feedback control loop has brought new challenges that were not encountered in traditional control systems. Important issues for NCSs include communication delays, control quality, stability, and safety (Sakthivel et al., 2015; Li Z. et al., 2016; Hu et al., 2017; Yaseen and Bayart, 2018). To diminish the deleterious network-induced side effects of an NCS, two lines of study have concentrated on the “control of network” and “control over network” principles (Gupta and Chow, 2010). The direction of “control of network” focused on investigation of network technology for data transmission (e.g., a wireless communication network), while the direction of “control over network” encompasses studies dealing with network delay and package drop, bandwidth allocation and scheduling, network security, fault-tolerant control, and integration of components with a suitable OS and interface (Gupta and Chow, 2010). In one study, modeling and analysis of network delay were carried out and methods of delay compensation were developed to deal with this problem (Kuzu et al., 2016). Significant progress in enhancing the stability and safety of the NCS has also been achieved in several studies. Bouazza and colleagues used a dynamic output feedback controller to improve the stability of a class of non-linear discrete-time NCS, while another group lead by Li adopted a novel delay-partitioning approach allowing the full consideration of the information on both the range of network-induced delays and the maximum number of consecutive data packet dropouts, which finally led to improved NCS stability (Bouazza, 2015; Li Z. et al., 2016). Hu et al. (2017) addressed the mean square stabilization for an NCS in case of a DoS type of cyber-attack. Minimal switching rules as well as methods for computing the state-feedback controllers were proposed to cope with this problem. Because the stability and safety of bioreactor control systems is a vital objective of bioprocess engineering, these studies on NCS stability and safety will further promote the implementation of NCSs in bioreactor control.

The FOCS for bioreactors, like the FCS and rising NCS, exhibits many important advantages over the classic HSCS for bioreactors (Table 1). First of all, the FCS and NCS have a two-layer physical structure or fully distributed flat structure, which is simpler than the three-layer structure of classic bioreactor HSCSs. Consequently, process parameters monitored by different sensors can be processed *in situ*, and orders are directly sent to paired actuators. Reducing the number of layers in the FOCS and the application of fieldbus systems or networks have reduced the complexity of wiring and the burden of communication between different layers. Furthermore, the dysfunction of one smart sensor does not interfere with the signals of other process parameters, which makes diagnosing process errors more convenient than in the HSCS for bioreactors. Smart sensors and actuators from different manufacturers that are compatible with the same fieldbus and network communication standards are also interchangeable, which may reduce the maintenance costs. Furthermore, the application of mobile terminals connected to the central computer instead of many touchscreens and keyboards on industrial field computers in the plant may also reduce the total capital investment. Researchers have also revealed the economic competitiveness of FCS compared with the classical HSCS for bioreactors (Rezabek, 2001).

### Knowledge-Based Control System

**FIGURE 4:**
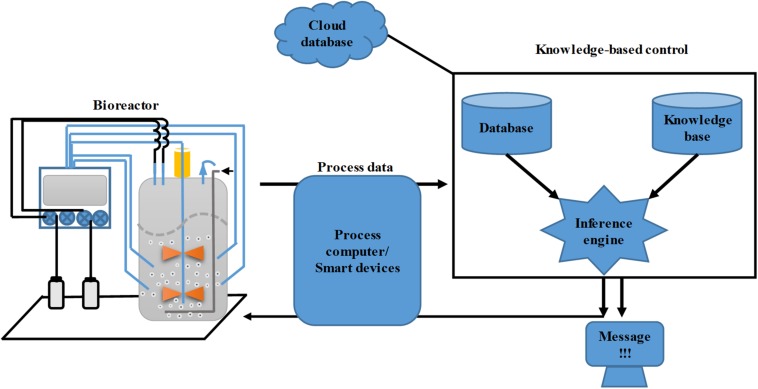
Representative structure of a knowledge-based control system for bioreactors.

With the development of process monitoring capacity through the application of novel sensors and on-/off-line PAT, integration of multiple on- and off-line process datasets into a real-time control system has become a major challenge in the development of bioreactor control systems. However, the development of FOCSs has partially solved the problem. Furthermore, very large amounts of historical bioprocess data have been accumulated in the bioindustry, and successful utilization of this digital resource calls for more intelligent control systems. KBCSs have emerged and ripened during the last decade, with the aim of solving this problem (Rathore et al., 2017; Borchert et al., 2019; Steinwandter and Herwig, 2019; Steinwandter et al., 2019). The KBCS has been implemented at two levels. The primary level entails the direct control of a specific process parameter via fuzzy logic, also known as fuzzy control (Wang et al., 2018). The higher level of implementation entails a knowledge-based supervisory control system that contains several modules (Figure 4) (Kohout et al., 2015). The first core module is the knowledge base, which was built upon the results of precise correlations of microbial physiology and biochemical measurements, including the knowledge of bioprocess experts and operators. The obtained linguistic rules are then transformed into computer-readable instructions. These rules are stored in the knowledge base of the control system. The second module is the database, which encompasses many different types of data, including the physical, chemical and physiological data of microbes inside the fermenter and the status of process monitoring and actuation devices, as well as the historical data of previous bioprocesses. The third module is the inference engine, which acts as the logical computation unit for process data quality evaluation, facility status diagnosis, physiological status prediction, control strategy, and indication message output (Kohout et al., 2015). During the development of the KBCS, classical control strategies like PID and model-based control have also been incorporated with fuzzy control to obtain better control performance. To date, many studies have demonstrated the successful applications of the KBCS.

Currently, there are already many applications of the KBCS in bioprocess control. A great number of applications of fuzzy control have been reported in wastewater treatment. For example, Wang et al. (2018) reported the design of a multivariable fuzzy controller to solve the control problem of vegetable waste fermentation. They constructed a structural matrix of fuzzy logic to convert the complex fuzzy logic into a simpler matrix operation, and they also adopted a new algorithm based on the least in-degree method to solve the problem of incomplete and inconsistent control rules. There are also reports of successful applications of higher-level knowledge-based supervisory control systems. Kohout et al. (2015) used knowledge-based control in antibiotic fermentation. In this work, a decision support system based on previous knowledge of the process was developed to support actions performed by process operators, which brought improvements in antibiotic production and reduced process variation. Birle et al. (2016) reported the application of an intelligent expert system based on fuzzy logic theory to control the yeast fermentation process. In this work, they introduced negative rules into the knowledge-base to mimic the human decision-making process. The incorporation of negative rules led to much more stable and accurate control of the process compared to the controller using only positive rules. Furthermore, the application of the KBCS has not been restricted only to process control. Together with model-based methods, Kroll et al. (2017) and Rajamanickam et al. (2017) reported the application of KBCS in the DOE for bioprocess development. From a broader perspective, the concept of bioprocess control and the functions of bioreactor control systems have been extended to the quality control and data management of the whole lifecycle of production. To solve bottleneck problems in the biotechnology industry, such as the long duration and capital investment requirements of new product development, the scale-down and scale-up effects of bioprocesses, scarcity of skilled workers, and incomplete knowledge about the real-time status of a production process, Steinwandter and Herwig reported the incorporation of ideas and methods from data science into bioprocess control, which will further stimulate the development and utilization of KBCSs (Steinwandter et al., 2019; Steinwandter and Herwig, 2019).

KBCSs for bioreactors still have a long way to go to fulfill their full envisioned functionality, as many critical problems have not yet been solved. The effectiveness of a KBCS depends on the quality of the knowledge database. However, there seems to be no golden standard for the construction of such databases. The functionality of KBCSs from different providers may be different and is still difficult to evaluate objectively. Due to the complexity of different bioprocesses, a knowledge database optimal for one plant/bioprocess may not be applicable to another plant/bioprocess, and specific rule sets need to be developed for each specific bioprocess. Another challenge stems from the inference engine, which has to evolve quickly in order to catch up with the ever-growing computation requirements of real-time process control. The development of AI, machine learning and process biochemistry, as well as further studies on the physiology of the utilized microorganisms, will lead to new strategies for solving these problems and strengthen the scope and effectiveness of KBCSs for bioreactors.

## Application of Smart Sensors and Actuators in Novel Bioreactor Control Systems

### Developing Novel Sensors and Cutting-Edge PATs as the Basis of Smart Sensors

Sensors have played indispensable roles in the above-mentioned control systems for bioreactors. Although most of the physical and chemical process parameters can be directly monitored with specific functional sensors, there are still some process parameters, especially those related to the physiological and biochemical characteristics of cultures, that cannot be easily measured. Various novel sensors and cutting-edge PATs have been developed as the basis of smart sensors, as has been discussed in some excellent reviews(Randek and Mandenius, 2018; Bockisch et al., 2019). Among them, studies on optical sensors and spectrographic analytical techniques have attracted a great deal of attention. As cell morphology is an important physiological phenotype of cultures that may have an important impact on the final products, many sensors and PATs have been developed to monitor these process parameters (Lemoine et al., 2017). In one study, Kuystermans et al. (2016) reported the application of flow cytometry-based methods for the monitoring of different apoptosis stages of mammalian cell cultures during a production process. Flow cytometry-based methods have also been used to study the morphology and viability of filamentous fungus *Penicillium chrysogenum* cultures and the population heterogeneity in response to changes in substrate availability in *Escherichia coli* and *Saccharomyces cerevisiae* chemostats (Heins et al., 2019; Veiter and Herwig, 2019). *In situ* microscopy is another effective tool for studying population heterogeneity. In one study, Marba-Ardebol et al. (2018) reported the real-time monitoring of the budding index in *S. cerevisiae* batch cultivations with *in situ* microscopy. Population heterogeneity caused by inhomogeneous culture conditions is a common phenomenon in many bioprocesses, and this heterogeneity is not only reflected in cell morphology but also includes differences in the intracellular concentrations of important metabolic intermediates (Heins and Weuster-Botz, 2018). Monitoring important metabolites such as lipids, proteins, and starch inside the cells is an urgent requirement in bioprocess development and large-scale production. Morschett et al. (2016) developed an automatic Nile red staining assay that enabled the high-throughput quantification of microalgal lipid production for bioprocess development. Several groups also reported the implementation of non-invasive Raman spectroscopy for the monitoring of mAb and lipid production during bioprocess development (He et al., 2017; Santos et al., 2019). Non-invasive Raman spectroscopy has also been applied for monitoring important parameters such as glucose concentration in a bioreactor for feedback control (Rowland-Jones and Jaques, 2018; Hirsch et al., 2019). Sensors based on fluorescence spectroscopy are widely used for different applications in bioprocess monitoring, and some detailed reviews have covered this topic (Faassen and Hitzmann, 2015). In one study, Janzen et al. (2015) evaluated two novel fluorimetric pH sensors for bioprocess monitoring at low pH, which extended the working range of previous fluorimetric pH sensors. Ladner et al. (2016) and Assawajaruwan et al. (2017) reported the integrated application of fluorescence spectroscopy with the microtiter plate cultivation system and the development of multi-wavelength (2D) fluorescence spectroscopy techniques for bioprocess monitoring. In addition, many different optical sensors and analytical techniques based on visible light (e.g., RGB sensors), UV light, or NIR, Raman, or pulsed terahertz spectra have also been developed for monitoring the biochemical parameters of the fermentation broth (Classen et al., 2017; Bockisch et al., 2019). These novel sensors and PATs could be further developed into smart sensors for application in the newly developed next-generation bioreactor control systems.

Developing advanced sampling methods for the on-line measurement of process parameters that currently cannot be monitored using online sensors is another important avenue of research on bioprocess control. FIA systems coupled with biosensors have provided a new strategy for monitoring the concentrations of important metabolites (Kumar, 2011). More recently, advanced analytical facilities have also been integrated with automatic sampling systems for bioprocess monitoring. In one study, Wu and Wee (2015) incorporated an automatic amino acid sample preparation protocol to a μSI system connected to an UPLC system for real-time, on-line amino acid separation and quantitation. In another recent study, Capozzi et al. (2017) successfully developed a methodology based on coupling a PTR-ToF-MS, an automated sampler, and tailored data analysis tools to monitor volatile organic compounds in the fermentation broth. High throughput cultivation is an important requirement of bioprocess development and strain evaluation. Along with the development of high-throughput process monitoring devices, the application of automatic high-throughput sampling platforms combined with fast analysis techniques is another way to get bioprocess data quickly. In one study, Cruz Bournazou and colleagues introduced an integrated robotic mini bioreactor platform for automated, parallel microbial cultivation with online data handling and process control (Haby et al., 2019). Through this platform, they have successfully combined high-throughput cultivation with fast high-throughput process analysis for process development. Development and application of those novel sensors and PTAs have enabled effective monitoring and control of bioprocesses ranging from normal cultivation in single bioreactors to high-throughput cultivation in multiple miniature bioreactors, from micro-liter-scale cultivation to industrial-scale bioproduction. The development of novel sensors and cutting-edge PATs is striving to catch up with the pace and facilitate the fast expansion of bioprocess applications in various circumstances. With the technical progress in diverse fields and the intersection of multiple disciplinary investigations, the development of novel sensors and PATs has stepped into the stage of the integration of intelligent data processing capability.

### Integration of Signal Processing, Actuation, Computation, and Communication Into Smart Sensors and Actuators

**FIGURE 5:**
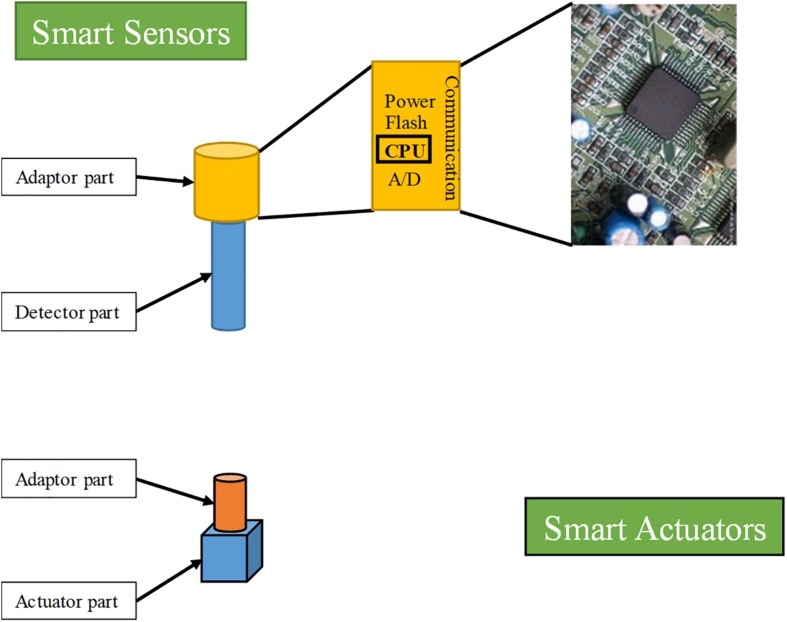
General architecture of a smart sensor and actuator.

As a foundation of the FOCS for bioreactors, smart sensors and actuators play critical roles in process parameter monitoring, signal processing, *in situ* computation, communication, and the execution of actuation instructions. The development of smart sensors and actuators is a very important stage in the evolution of bioreactor control systems. The general architecture of smart sensors and actuators for application in bioprocess monitoring and control is shown in Figure 5 (Gary et al., 2010). The basic architecture of smart sensors for bioprocess monitoring and control comprises a classic sensor unit and an adapter unit. The function of the sensor unit in a smart sensor is monitoring and transforming process parameters into the form of analog signals, the same as that of traditional sensors. The functions of the communication and computation component (adapter unit) in a smart sensor consist of A/D and D/A transformation of signals, conditioning and self-diagnosing sensors, control algorithms run by the internal microprocessors, sending and receiving data from other smart sensors/actuators or the central computers, and power supply. The smart sensor performs a part of the functions of the industrial field computers in the classic HSCS for bioreactors. As the computational tasks of specific smart sensors are usually less taxing than those of industrial field computers, the requirements for computational capacity are also less demanding. With the fast development of cheaper and smaller hardware platforms, functional OSs, power-supplies, and micromanufacturing techniques, the application of smart sensors in various fields is increasing (Lee et al., 2015; Zhao et al., 2016; Perez et al., 2018; Zhang et al., 2018).

To date, smart sensors have found wide-ranging applications in healthcare, smart driving, smart cities, industrial plants, and IoT networks, as well as in bioreactor control (Lee et al., 2015; Hernandez-Rojas et al., 2018; Perez et al., 2018; Zhang et al., 2018). In one study, Lee et al. (2015) introduced a RFID-based wireless smart-sensor system, composed of a Pt_rGO RFID sensor tag and an RFID-reader antenna-connected network analyzer, to detect hydrogen gas. This sensor has wide application prospects in environmental gas detection, including bioprocess monitoring and control (Lee et al., 2015). In a more recent study, Perez et al. (2018) introduced a smart sensor designed to monitor cell growth in a micro-well. The investigated cell culture served as a “biological oscillator” for extracting signals of cell growth and cell number. Zhang et al. (2018) reported the design, fabrication, and implementation of an array-type MEMS piezoresistive intelligent pressure sensor system to meet the requirements of high sensitivity and linearity of monitoring in a radiosonde. The application of smart sensors under these circumstances has distinct advantages, including more consolidated solutions for process monitoring, simultaneous monitoring of large numbers of process parameters, and high efficiency of the sensor network.

Research on smart sensors has also become a hot topic in electronics and computer science, with profound effects on many application fields. Incorporation of communication and computation components into the smart sensor system has led to increased energy demand, so that a stable and efficient power supply has become a critical issue in smart sensor development (Lin et al., 2019). In many application circumstances, a wireless sensor network has become a superior choice (Zhu et al., 2018). Investigations dealing with the safety, efficiency, and stability of the communication network have also become a popular topic, and strategies such as setting common criteria from the beginning of smart sensor design have also been suggested to deal with this concern (Bialas, 2010a,b).

The concept of smart actuators has two different interpretations. In some studies, it refers to actuators made of “smart materials” that have intrinsic characteristics of built-in sensors, actuators, and control mechanisms in their microstructures. In the second case, it refers to an intelligent instrument (like a DC motor or valves) that serves as an actuator in process control (Barasuol et al., 2018). Here, we will discuss smart actuators according to the second definition. According to the very early definition given by Staroswiecki and Bayart (1996), the typical functional structure of smart actuators consists of components for communication, management (including data input, validation, elaboration, and database management), decision, and action. However, the concrete structure of smart actuators for specific applications may be different. As an essential node of the FOCS for bioreactors, smart actuators are envisioned to be capable of receiving signals from smart sensors or controllers and responding to these instructions for process control. In most cases, the actuators and the sensors are physically separated, such as those used for the monitoring and control of pH, temperature, and DO. Wireless communication is preferable to protecting a cable in a harsh environment. In other circumstances, the actuators and sensors are physically integrated, such as those for the monitoring and control of rotation speed. In such cases, interactions between sensors and actuators are more convenient.

Smart actuators have been widely used for the control of vehicles and robots and in the biotechnology industry (Perotti et al., 2006; Van Loon, 2015; Barasuol et al., 2018). Perotti et al. (2006) reported a VHM smart actuator for a liquid hydrogen storage system, with emphasis on its self-diagnosing function. Van Loon (2015) reported that the application of smart valves controlled by an FCS in a beverage plant increased the reliability and efficiency of its production line. As valves are important functional parts of bioreactors and related pipelines, the application of smart actuators such as the VHM system can potentially greatly increase the stability and controllability of bioprocesses. As smart actuators are generally used in larger systems, their proper functioning has vital consequences for fulfilling the higher-level production goals such as yield, byproduct profiles, and, ultimately, the economics of the bioprocess.

### Multi-Parameter Sensors for the Simultaneous Monitoring of Multiple Process Parameters

Multi-parameter sensors are another interesting direction of smart sensor development (Bockisch et al., 2014, 2019; Sardesai et al., 2015). In most cases, many process parameters need to be monitored simultaneously for process development, and the application of multi-parameter sensors could dramatically reduce the total number of sensors used. Multi-parameter sensors have been developed for applications in many fields. Eder et al. (2014) developed a CMOS sensor for combined temperature and humidity measurements of micro-packages for implanted integrated circuits to ensure their safe operation. Wang et al. (2014) reported the development of a smart sensor that could simultaneously measure the reflectance index and temperature of tested samples, with possible applications in chemical and biological investigation. Bockisch et al. (2014) reported the application of a mobile multi-parameter sensor for the *in situ* investigation of the liquid phase in industrial yeast fermentations. Sardesai et al. (2015) reported the development of a composite optical detector that can be used for the simultaneous measurement of pH and DO.

**FIGURE 6:**
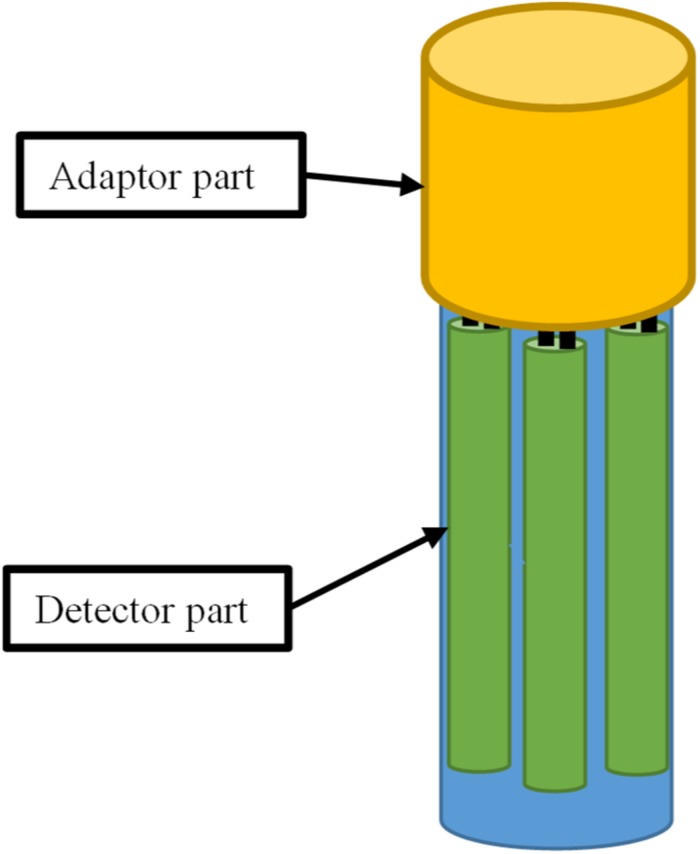
Example of the architecture of a multi-parameter sensor for simultaneous monitoring of multiple process parameters.

Currently, most of the sensors used in bioreactor control systems were designed to measure a single parameter. Consequently, many openings on the top or wall of the fermentation bioreactor are required, which makes them more complicated to manufacture and more expensive, increases the risk of contamination, and adds to the inconvenience of operation. Conversely, the application of multi-parameter sensors designed for the simultaneous monitoring of multiple parameters can greatly reduce the total number of sensors used in bioreactors, thus requiring a smaller number of openings during the manufacturing process, improving safety and resistance to contamination. A possible design of a multi-parameter sensor is depicted in Figure 6. For example, the pH, temperature, and DO measurement functions can be integrated into one smart sensor. An adapter for the multi-parameter sensors specifically for signal processing and communication could also be developed. Spectroscopic analysis including NIR, Raman, and fluorescence spectra also provide an alternative strategy for developing novel multi-parameter sensors (Wang et al., 2014). The application of multi-parameter smart sensors could greatly increase the integration of control systems and simplify the workflow for the workers.

## Expanding the Application of Cutting-Edge Bioreactor Control Systems in Various Bioprocesses

### Parallel Miniature Bioreactors for Bioprocess Development

Parallel miniature bioreactors with small working volumes (also called microbioreactor systems) are an important alternative to the traditional batch cultivation apparatuses for use in bioprocess development and scale-up/scale-down investigation. Many important studies offer a detailed discussion of this topic, and many commercialized products (e.g., DASGIP from Eppendorf, AMBR from Sartorius Stedim Biotech) are also available in the market (Faust et al., 2014; Hemmerich et al., 2018a; Bjork and Joensson, 2019; Sandner et al., 2019; Znidarsic-Plazl, 2019). Process monitoring and control of miniature bioreactors are more complicated than single-bioreactor control, but investigations have shown encouraging results. From a structural perspective, parallel miniature bioreactors could be grouped into two categories, the microplate-, microchamber-, and microfluidics-based miniature bioreactors, and the stirring tank-based miniature reactors (Hemmerich et al., 2018a). For the microplate-, microchamber-, and microfluidics-based miniature bioreactors, monitoring and control systems based on optical sensors are very effective choices. For example, Szita et al. (2005) reported the development of a multiplexed microbioreactor system for high-throughput bioprocess investigation, using a group of optical fiber sensors for the simultaneous monitoring of OD, DO, and pH. Notably, very similar process parameter curves were observed compared with a control system. On-line Raman spectroscopy and design-of-experiment strategies have also been adopted to monitor multiple metabolic and physiological parameters of cultures in parallel miniature bioreactors (Rowland-Jones and Jaques, 2019). Furthermore, the integration of miniature bioreactors with robotic sampling devices, fast PATs, and repetitive small volume sampling has also been used for efficient phenotyping of cultures in microplates, microchambers, and microfluidic devices (Morschett et al., 2017; Hemmerich et al., 2018b). The incorporation of design-of-experiment programs into the control system and knowledge-based process modeling have also been used to facilitate bioprocess development using a group of miniature bioreactors (Moller et al., 2019). In parallel stirred-tank bioreactors, only a limited number of parameters can be monitored, and new methods need to be developed for these bioprocesses. For example, Frachon et al. (2006) developed a multiple microfermenter battery, each with a working volume of 80 ml, for automated parallel cultures of microorganisms producing recombinant proteins and the optimization of the cultivation protocols. Three miniature probes were used for parameter monitoring. In the parallel miniature bioreactors, process parameter monitoring and control using traditional sensors, actuators, and the HSCS leads to a very limited operation space on the top of the bioreactors, increasing the inconvenience of operation and the risk of contamination. Application of FOCSs for bioreactors with multi-parameter smart sensors and actuators will facilitate the operation and maintenance of parallel miniature bioreactors. As the most important application of miniature bioreactors has been cell culture and process evaluation, in addition to the normal control and data gathering function of bioreactor control systems, the requirements of design-of-experiment, data evaluation, and analysis capacities make the development of KBCSs of miniature bioreactors an important research topic (Abt et al., 2018).

### Continuous Culture Bioreactors for Frontier Multidisciplinary Investigation

Life science investigation is an important task in space exploration, and it stands at the frontier of multidisciplinary investigation, which has attracted a great deal of attention from many countries. For example, continuous cultivation of microalgae in an illuminated bioreactor has become an extremely attractive idea for O_2_ regeneration and control in an emergency in the BLSS in space exploration. Stable and effective monitoring and control of this complicated system also dependents on a robust bioreactor control system (Hu et al., 2012). The ability of microalgae to convert sunlight and carbon dioxide into chemical energy has also attracted a lot of attention from researchers from both academia and industry. These organisms can produce a huge variety of products, such as high-value proteins, pigments, fatty acids, and biofuels. Research on the growth kinetics of microalgal and suitable control strategies in different photobioreactors have been the topics of many important studies (Koller et al., 2017; Pfaffinger et al., 2019). Application of FOCSs for bioreactors based on smart sensors and actuators could reduce the complexity of the required hardware, which would, in turn, increase the robustness of the autonomous control system. Based on the FOCS for bioreactors, long-term continuous cultivation and more complex cultivation modes could be carried out automatically with a lower dependence on human interference. ALE is an effective approach for the systematic optimization of strain phenotypes and has been widely applied in biotechnology. Traditional ALE experiments require constant human operation. In one long-term evolution experiment, two populations of *Escherichia coli* B were adapted to a glucose minimal medium for 10,000 generations, which lasted many years (Lamrabet et al., 2019). Recently, Wong et al. (2018) developed a bioreactor termed “eVOLVER” with an FOCS for precise and automated control of growth conditions for high-throughput cultivation of yeast and bacteria. Smart actuators were used to control the growth temperature via a PID-controlled heater, and an Ethernet network was used for data transmission between different modules of the control system to fulfill functions of vial-to-vial cell transfer and exchange of culture medium. Automation of ALE experiments supported by bioreactors with an FOCS has made this process more efficient and reproducible.

## Summary and Perspectives

In conclusion, novel FOCSs for bioreactors including advanced FCSs and NCSs based on smart sensors and actuators represent an advanced stage of bioprocess control compared with the classical HSCSs for bioreactors. They have the advantages of high efficiency, high interoperability, and stability, low maintenance cost, and advanced management capability, even though drawbacks still exist. In the future, the development of FOCSs for bioreactors offers great opportunities. From one perspective, the fast progress in the field of information and communication technology (e.g., 5G and IoT), mechanical engineering, and frontier research in basic science will further promote the development of smart sensors/actuators, communication channels, and other components of the control system. Thus, bioreactor FOCSs may continuously evolve to a more advanced stage. From another perspective, modern biotechnology has stepped into the era of synthetic biology and systems biology, which has provided new ideas, tools, and methods to further understand important factors that influence bioprocesses. With the deeper understanding of specific bioprocess reaction mechanisms and growing knowledge of the process dynamics, bioreactor KBCSs based on powerful computation capacity through AI and cloud computation will become more effective and reliable. This, in turn, may further stimulate the optimization of bioreactor FOCSs and benefit bioprocess control.

## Author Contributions

BW conceived the work, investigated related topics, and wrote the manuscript. ZW, TC, and XZ discussed the topic and edited and refined the manuscript. All authors approved the publication of this work.

## Conflict of Interest

The authors declare that the research was conducted in the absence of any commercial or financial relationships that could be construed as a potential conflict of interest.

## Abbreviations

μ SI: micro sequential injection
A/D: analog/digital
AI: artificial intelligence
ALE: adaptive laboratory evolution
BLSS: Bioregenerative Life Support System
CMOS: complementary metal-oxide semiconductor
D/A: digital/analog
DCS: distributed control system
DIY: do-it-yourself
DO: dissolved oxygen
DOE: design of experiment
DoS: denial of service
FCS: fieldbus control system
FF: Foundation Fieldbus
FIA: flow injection analysis
FOCS: flat organizational control system
HSCS: hierarchical structure control system
IoT: Internet of Things
KBCS: knowledge-based control system
MES: manufacturing execution system
MEMS: microelectromechanical system
NCS: networked control system
NIR: near-infrared
OD: optical density
OS: operating system
PAT: process analytical technique
PTR-ToF-MS: proton transfer reaction with a time-of-flight mass spectrometer
PID: proportional–integral–derivative
PLC: programmable logical controller
Pt_rGO: Pt-decorated reduced graphene oxide
RFID: radio frequency identification
UPLC: ultra-performance liquid chromatography
VHM: valve health monitor.
